# Systematic analysis of the prognosis and immune infiltration of E2Fs in thyroid carcinoma

**DOI:** 10.3389/fgene.2023.1215984

**Published:** 2023-07-25

**Authors:** Xinghao Fu, Xing Zhou, Feihong Ji, Qi He, Xinguang Qiu

**Affiliations:** ^1^ Department of Thyroid Surgery, First Affiliated Hospital of Zhengzhou University, Zhengzhou, Henan, China; ^2^ Department of Plastic and Aesthetic Surgery, Henan Provincial People’s Hospital, Zhengzhou, Henan, China

**Keywords:** E2F transcription factors, thyroid carcinoma, The Cancer Genome Atlas, prognosis, immune infiltration

## Abstract

**Purpose:** To evaluate the diagnostic and prognostic utility of E2F transcription factors (E2Fs) in thyroid carcinoma (THCA) and their association with immune infiltration.

**Methods:** The transcription and protein levels of E2Fs in THCA tissues were examined using the R language and the Human Protein Atlas (HPA) database in this research. We utilized the UALCAN and GEPIA2 databases to analyze the association between the level of E2Fs and the clinicopathological features of THCA. The prognostic utility of E2F expression in THCA was studied using the R language and the Gene Set Cancer Analysis (GSCA) database. Over-representation analysis (ORA) and gene set enrichment analysis (GSEA) were employed to analyze the effect of E2F family members. The TISIDB database and Tumor Immune Estimation Resource (TIMER) database were utilized to investigate the relationship between E2F expression and the level of immune infiltration in thyroid cancer.

**Results:** E2Fs are highly conserved in thyroid carcinoma and rarely mutated. E2Fs are strongly expressed in THCA and are highly related with the clinicopathological stage of THCA. Patients with THCA have a poor prognosis when E2Fs are highly expressed. The function of E2Fs in THCA may be closely related to the renin–angiotensin system (Ras) signaling pathway, platelet-derived growth factor (PDGF) signaling pathway, apoptosis, and immune response. With regard to the immune infiltration, E2F expression and tumor-infiltrating lymphocytes exhibited a positive connection.

**Conclusion:** The level of E2Fs is connected with the prognosis and immune infiltration level in THCA, revealing that E2Fs may be a prognostic and immune infiltration cell marker in THCA patients.

## 1 Introduction

Thyroid carcinoma is the most prevalent endocrine cancer, which accounts for approximately 2.1% of all cancers worldwide. The incidence of THCA is increasing year by year worldwide, and it has become the fastest growing malignant tumor. However, its pathogenesis has not been fully elucidated (Goodarzi et al., 2019). THCA is usually characterized by infiltration of inflammatory immune cells. In recent years, many experimental and epidemiological sources of evidence have shown that immune infiltration is correlated with the emergence and progression of several malignant tumors including THCA (Liotti et al., 2012). In the thyroid gland, tissue-infiltrating immune cells are able to secrete cytokines, thereby participating in the emergence and progression of autoimmune inflammation and malignant tumors (Varricchi et al., 2019).

The E2F family is an extremely important group of transcription genes in organisms. It is named thus because it is considered to be a kind of cytokine activated by Ela when it was discovered and it is related to the activation of the adenovirus E2 promoter. However, eight members of the E2F family have been discovered in mammals, namely, E2F1–8 (Sozzani et al., 2006). E2Fs play an important role in cell proliferation, apoptosis, differentiation, senescence, deoxyribonucleic acid (DNA) damage response, and DNA repair (DeGregori and Johnson, 2006). Recently, research studies have found that E2Fs play a role in the formation and development of many malignant cancers through different mechanisms (Garneau et al., 2009; Fang et al., 2018; Moreno et al., 2021). However, the function of E2Fs in THCA remains unclear. So, we probed the association among E2F expression, prognosis, and tumor immunity in thyroid cancer using multiple databases.

Eight E2Fs in THCA were the subject of a detailed and thorough bioinformatic analysis in this study, and their potential as diagnostic and prognostic biomarkers was assessed based on several significant public databases. This study provides comprehensive data for clinicians to choose appropriate therapeutic targets and to more precisely predict the long-term prognosis of thyroid cancer patients. It may also help optimize immunotherapy for patients with THCA.

## 2 Materials and methods

### 2.1 E2F family expression data analysis

The data on the pan-carcinoma project were downloaded from TCGA database. With the use of the R language (version 3.63), the Mann–Whitney *U* test was utilized to examine the difference in expression among cancer and normal tissues, and the ggplot2 package was utilized for visualization.

In this study, immunohistochemical images of E2F1, E2F2, and E2F8 in THCA and normal tissues were identified and extracted from HPA (https://www.proteinatlas.org/) for comparison (Thul et al., 2017).

We used the GEPIA2 (http://gepia2.cancer-pku.cn/#analysis) database to study the expression profile of E2Fs in THCA and normal thyroid tissues and to investigate the association between their level and clinical stages of THCA patients (Tang et al., 2019).

The UALCAN database (http://ualcan.path.uab.edu/) is an established secondary site based on the analysis of TCGA database (Chandrashekar et al., 2017). In this study, the gene name of E2F family was first input and the cancer type was selected as thyroid cancer to study the level of the E2F gene family in THCA and its connection with clinicopathological features of THCA patients.

### 2.2 E2F family genetic alteration data analysis and interaction network construction

Appropriate THCA datasets were selected from the cBioPortal database (https://www.cbioportal.org/) to analyze the mutation rate of the E2F gene family in THCA (Cerami et al., 2012), to explore whether the expression changes of the E2F gene in THCA are caused by gene mutations, and to speculate the expression stability of E2Fs in THCA.

The TCGA thyroid cancer project’s Level 3 HTSeq-FPKM format RNAseq data were analyzed using the R language (version 3.63) to examine the expression association between eight E2F family members using Spearman’s correlation analysis, and the ggplot2 package was used for visualization.

The STRING database (http://stringdb.org) is used to assess and generalize data based on protein–protein interactions, including their functional and physical correlations (Szklarczyk et al., 2017). The E2F gene was input into the homepage of the official website of the GeneMANIA database (https://genemania.org/) to construct a dendrogram of its related gene network (Franz et al., 2018).

Using the online analytical tool VENN (http://bioinformatics.psb.ugent.be/webtools/Venn/), Venn diagrams were constructed to obtain the cross genes shared by the two gene datasets (Bardou et al., 2014).

### 2.3 Survival data analysis of the E2F family in THCA

In this research, we used the GSCA (http://bioinfo.life.hust.edu.cn/web/GSCALite/) online tool to investigate the association between prognosis and E2F family in various tumors (Liu et al., 2018).

The patients’ clinical information of 510 THCA tissues and 58 normal tissues were obtained from TCGA database. The clinical information of patients with THCA was integrated, and the Kaplan–Meier analysis with the log-rank test was performed using the survival package (version 3.2-10). The results from the survival analysis were visualized using the survminer package (version 0.4.9).

### 2.4 Functional analysis of the E2F family in THCA

In this study, the LinkInterpreter module of LinkedOmics (http://www.linkedomics.org/login.php) was used to perform ORA to explore the signaling pathways associated with the participation of the E2F family in THCA. Pathways with FDR ≤ 0.05 were considered significant (Vasaikar et al., 2018).

According to the median gene expression, tumor samples from TCGA project were divided into high expression and low expression groups. To identify core gene-related signaling pathways, c2.cp.kegg.v7.2.symbols.gmt was chosen as a reference using GSEA software for analysis (Subramanian et al., 2005). By examining 1000 permutations, the normalized enrichment analysis score (NES) was calculated. According to the previous research, |NES| ≥ 2.0, *p* < 0.05, and false discovery rate (FDR) < 0.25 were used as thresholds according to the standard screening gene set (Fu et al., 2022).

### 2.5 Relationship between E2F family expression and immunosuppressive agents in THCA

The TISIDB database (http://cis.hku.hk/TISIDB/) is one of the commonly used databases for cancer immune infiltration markers (Ru et al., 2019). E2F family members were input into the TISIDB database, and the association between their expression and that of immune checkpoint inhibitor genes was examined.

### 2.6 Relationship between E2F family and immune infiltration in THCA

The TIMER database (https://cistrome.shinyapps.io/timer/) is one of the commonly used cancer immune infiltration marker databases (Li et al., 2017). The correlation between E2F family and immune cells was explored using the gene module in the TIMER database. Subsequently, we used the somatic copy number alteration (SCNA) module to explore the relationship between tumor infiltration level and somatic copy number changes of the E2F family in THCA. Finally, the correlation analysis module was employed to investigate the association between E2F levels and immune-infiltrating cell markers.

### 2.7 Statistical analysis

Survminer, survival, and ggplot2 packages of R software (version 3.6.3) were utilized. The Mann–Whitney *U* test was utilized for differential expression analysis. Kaplan–Meier and log-rank sum tests were utilized for prognostic analysis. The statistical significance was set at *p* < 0.05.

## 3 Results

### 3.1 Analysis of E2F family expression data

**FIGURE 1:**
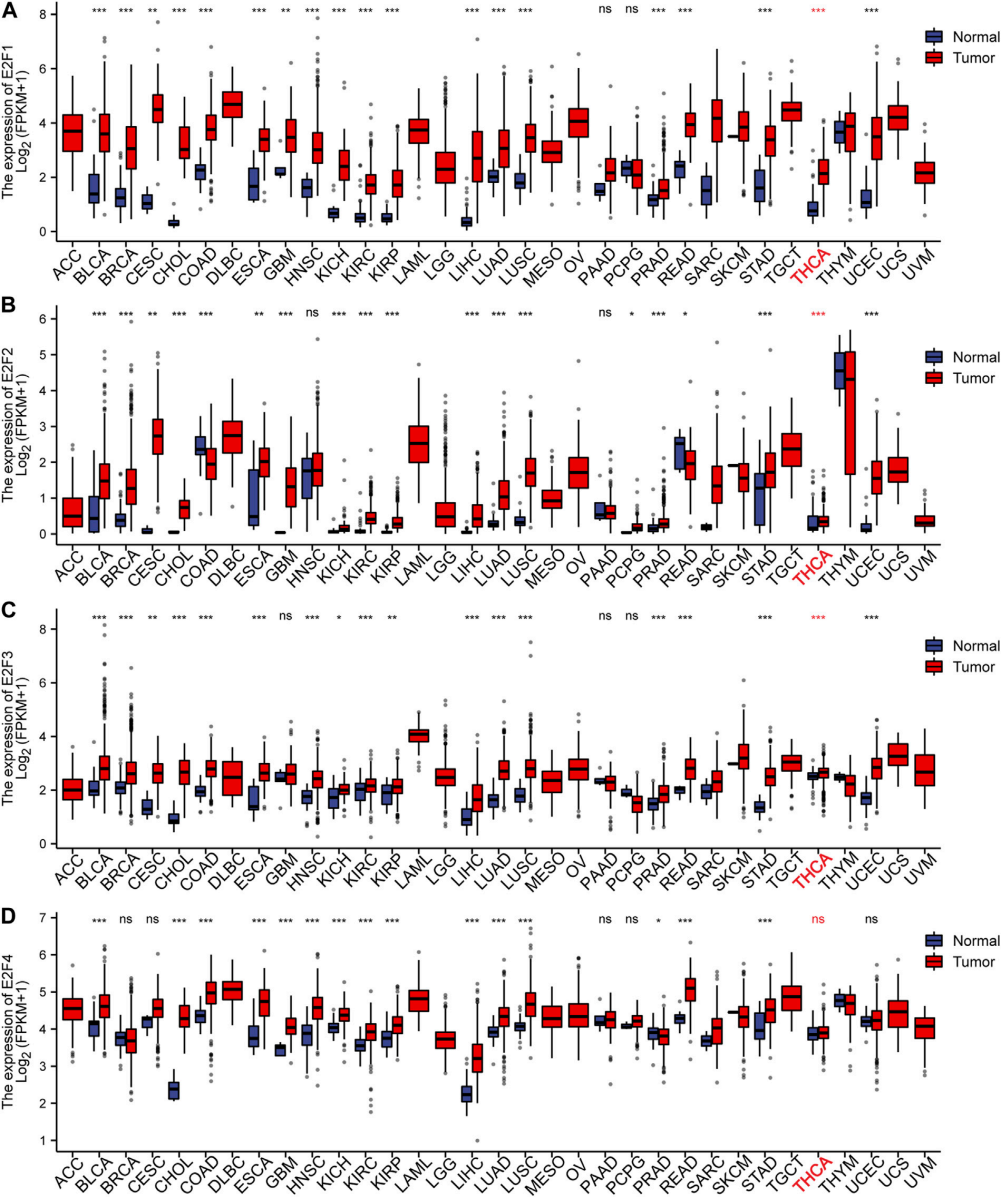
Expression of the E2F family in pan-carcinomas. **(A-D)** E2F1–4 expression in pan-carcinomas. *p < 0.05; **p < 0.01; ***p < 0.001.

**FIGURE 2:**
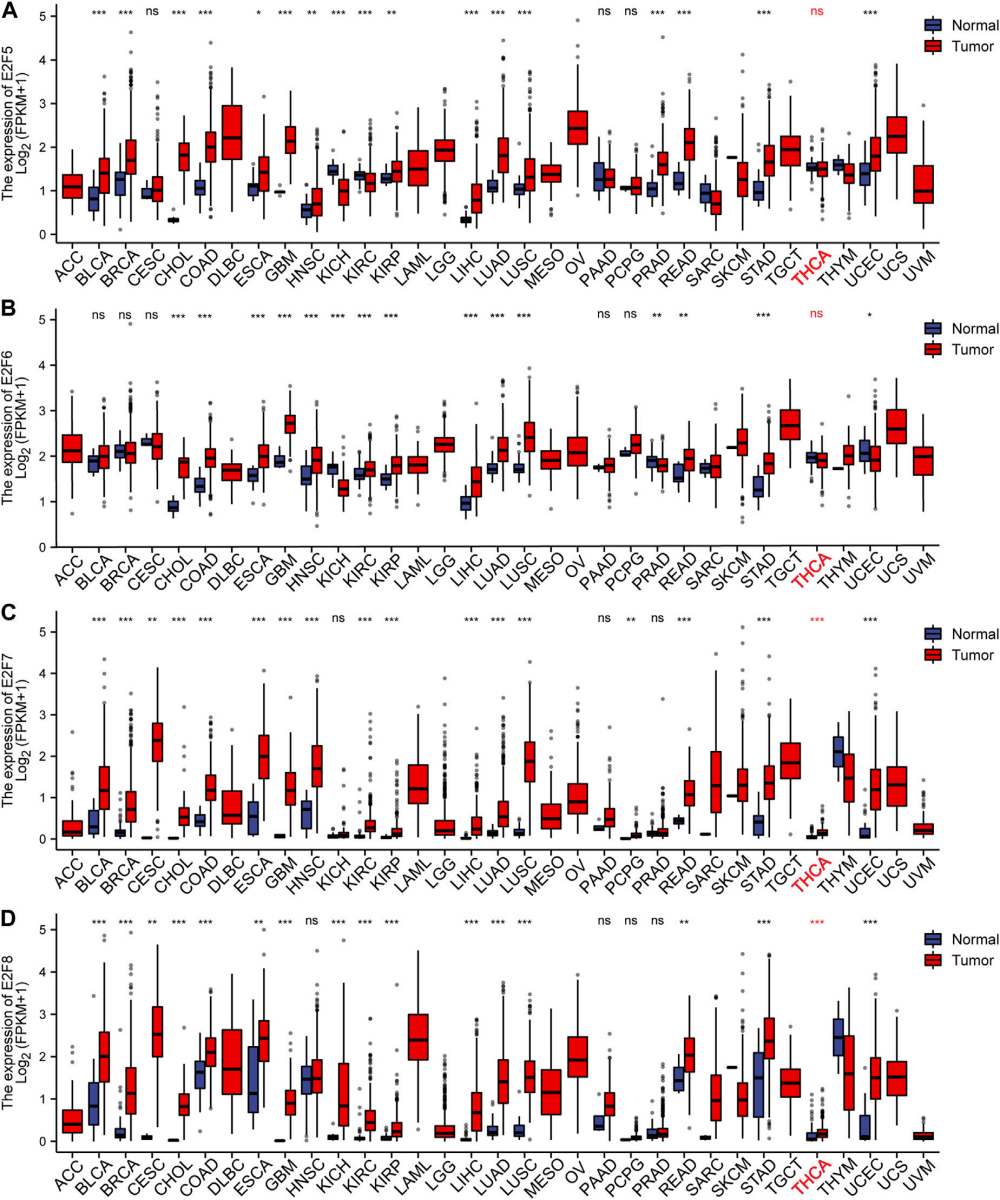
Expression of the E2F family in pan-carcinomas. **(A-D)** E2F5–8 expression in pan-carcinomas. *p < 0.05; **p < 0.01; and ***p < 0.001.

To study the levels of the E2F family in pan-carcinoma, RNAseq data from TCGA were obtained, and the results were analyzed and calculated by the R language and visualized by the ggplot2 package. The outcomes displayed that the E2F gene was greatly expressed in most cancers (Figures 1, 2). For THCA, E2F1, 2, 3, 7, and 8 genes were strongly expressed, indicating that the E2F family plays a significant carcinogenic role in THCA (Figures 1, 2). Next, we investigated the protein level of E2Fs in THCA. Immunohistochemical results of three E2F members, namely, E2F1, E2F2, and E2F8, in THCA were found in the HPA database. The outcomes displayed that the protein level of E2F1 was higher in THCA, while E2F2 and E2F8 did not significantly vary from normal tissues in terms of protein expression levels (Supplementary Figure S1). The findings of the analysis of the association between E2F transcription levels and THCA tumor stages using the GEPIA2 database displayed that the level of E2F2 and E2F5 was associated with the THCA cancer stage (Supplementary Figure S2). Finally, we discovered that the E2F gene family was related to race, gender, age, TNM stage, pathological stage, histological type, extrathyroidal extension, and primary neoplasm focus type in THCA (Supplementary Table S1).

### 3.2 Analysis of genetic alteration data of E2Fs and construction of the interaction network

**FIGURE 3:**
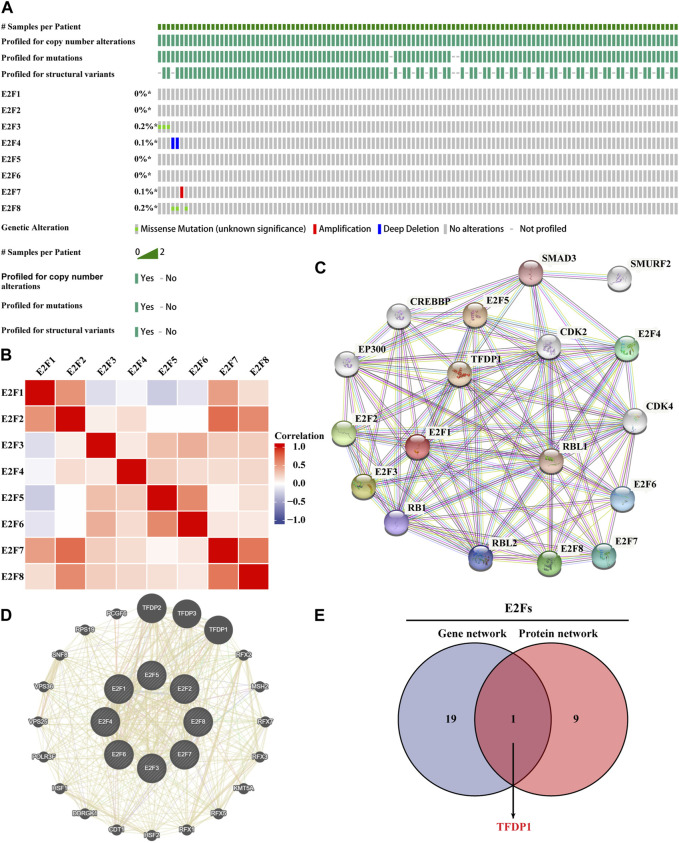
Genetic alteration data of the E2F family and construction of the gene–protein interaction network. **(A)** Overview of genetic alteration data of the E2F family in THCA. **(B)** Association of the E2F family members in THCA. **(C)** Network diagram of E2F family protein–protein interactions. **(D)** Network diagram of E2F family gene and intergene interactions. **(E)** Proteins and genes that interacted with the E2F family were cross-analyzed.

The genetic alterations of E2Fs in the TCGA-THCA samples were investigated using the cBioPortal database. The findings demonstrated that the E2F family was highly conserved in THCA and rarely mutated (Figure 3A). We then used the muTarget database to explore the expression of genes that is affected by the E2F mutation in thyroid cancer and genes themselves having mutations that affected the expression of E2Fs. Finally, only mutations in NRAS in thyroid cancer were found to reduce E2F8 expression. This is consistent with the highly conserved results of E2Fs in thyroid cancer. Then, we used the R language to perform correlation analysis to explore the expression association among the eight members of the E2F family in THCA. The ggplot2 package was used for visualization. The findings demonstrated that the vast majority of E2F family members were positively correlated (Figure 3B). TP53, a key gene of the apoptosis pathway, and AKT1, a key gene of the cell survival pathway, interact closely with E2Fs. Therefore, we explored the expression patterns between these markers and E2Fs. The results of the chord diagram showed that there was a significant positive correlation between them (Supplementary Figure S3). Finally, the gene and protein networks that potentially interacted with the E2F family were investigated using the GeneMANIA and STRING databases. Ten target proteins and 20 target genes were identified to interact with the E2F family (Figures 3C, D). Cross-analysis of the aforementioned two groups yielded a common molecule, TFDP1 (Figure 3E).

### 3.3 Survival data analysis of the E2F family in THCA

**FIGURE 4:**
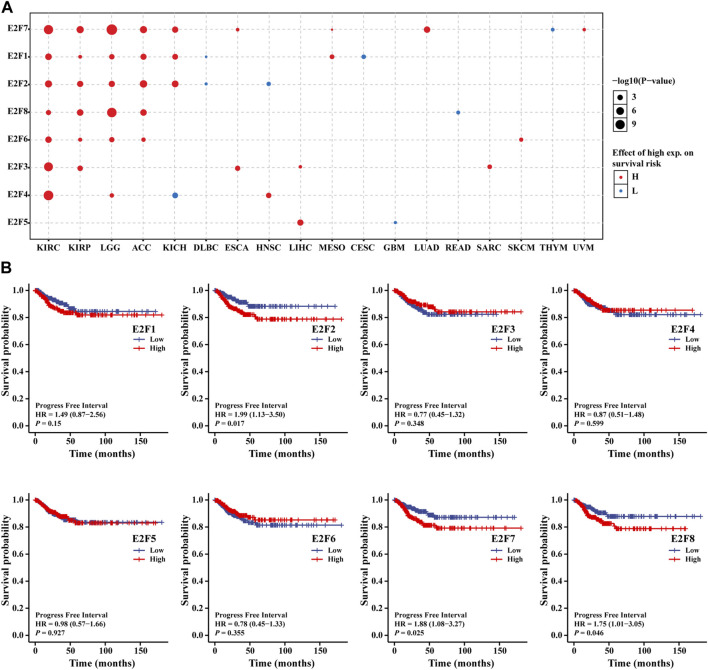
Prognostic analysis of the E2F family in THCA. **(A)** Relationship between the prognosis of tumors and the E2F family in pan-cancer. **(B)** Association between the E2F family members and PFI in THCA.

We divided tumor cases into two groups based on the level of expression of each E2F members; next, we utilized TCGA dataset to study the relationship between E2F gene level and THCA patients’ prognoses. A pan-cancer study utilizing the GSCA online tool revealed, as shown in Figure 4A, that the E2F family was associated with the prognosis of a range of malignancies and that the stronger the E2F family expression, the worst the prognosis. Next, the survival package was used to analyze the prognosis of RNAseq data of the TCGA-THCA project. As THCA has a better prognosis, a progression-free interval (PFI) was chosen instead of overall survival (OS) as the outcome event. The findings demonstrated that patients with high E2F2, E2F7, and E2F8 expression had shorter PFI (Figure 4B). The subgroup K–M analysis was then used to assess the association between the level of E2F members and clinicopathology of THCA in order to further understand the association between E2F family and clinical characteristics of THCA. We discovered that the high E2F1 level was associated with poor PFI in patients with THCA in age, T stage, and pathological stage (Supplementary Table S2). Finally, to investigate the independent risk variables of PFI in THCA, regression analyses were carried out. Univariate Cox analysis showed that T stage, M stage, pathologic stage, extrathyroidal extension, E2F2, E2F7, and E2F8 were the risk factors for PFI (*p* = 0.001, 0.001, 0.001, 0.023, 0.017, 0.025, and 0.046, respectively). Only M stage (*p* = 0.032), according to multivariate Cox regression analysis, was identified as an independent risk factor for PFI (Supplementary Table S3).

### 3.4 Functional analysis of E2F in THCA

**FIGURE 5:**
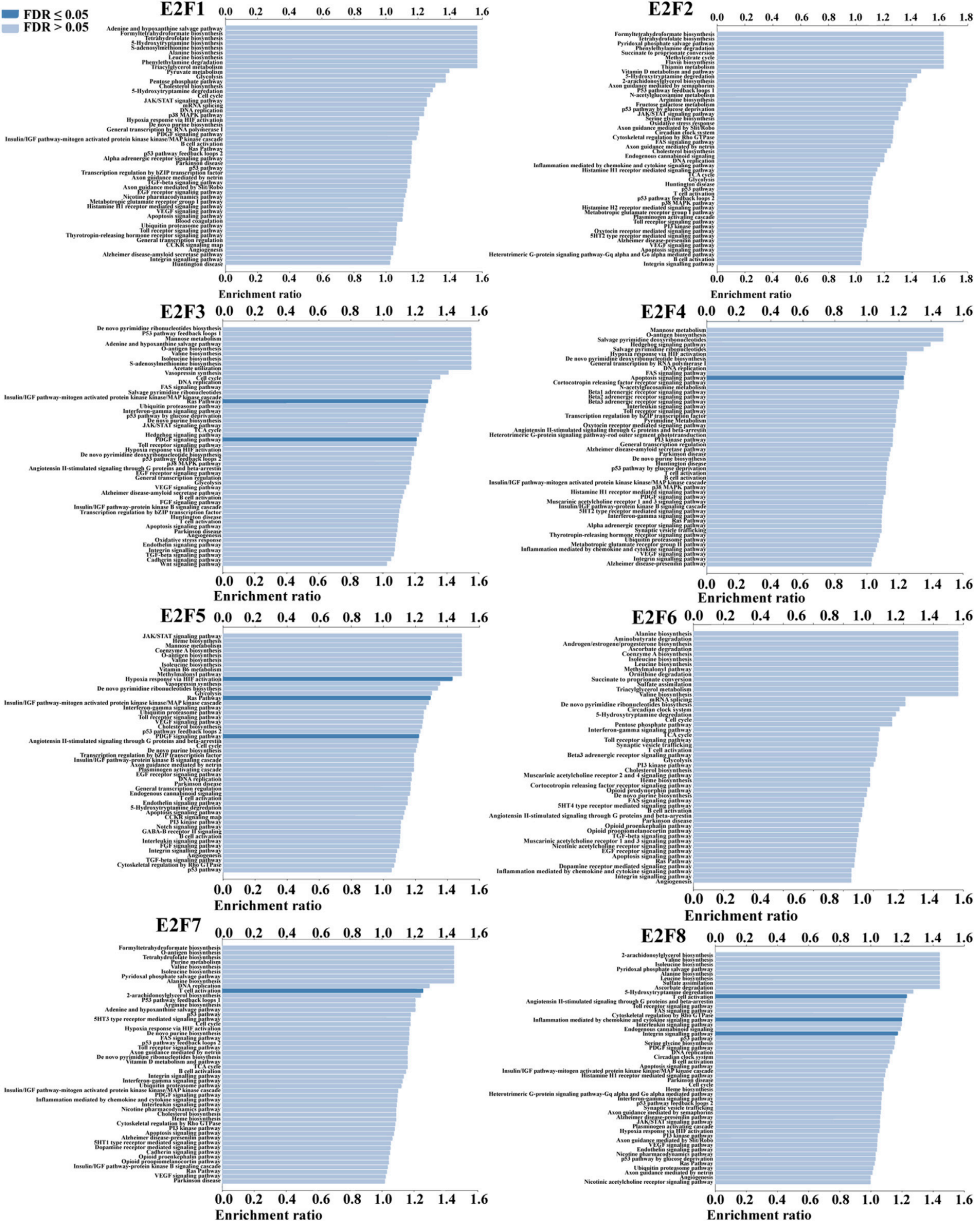
Results of ORA of the E2F family members in THCA.

**FIGURE 6:**
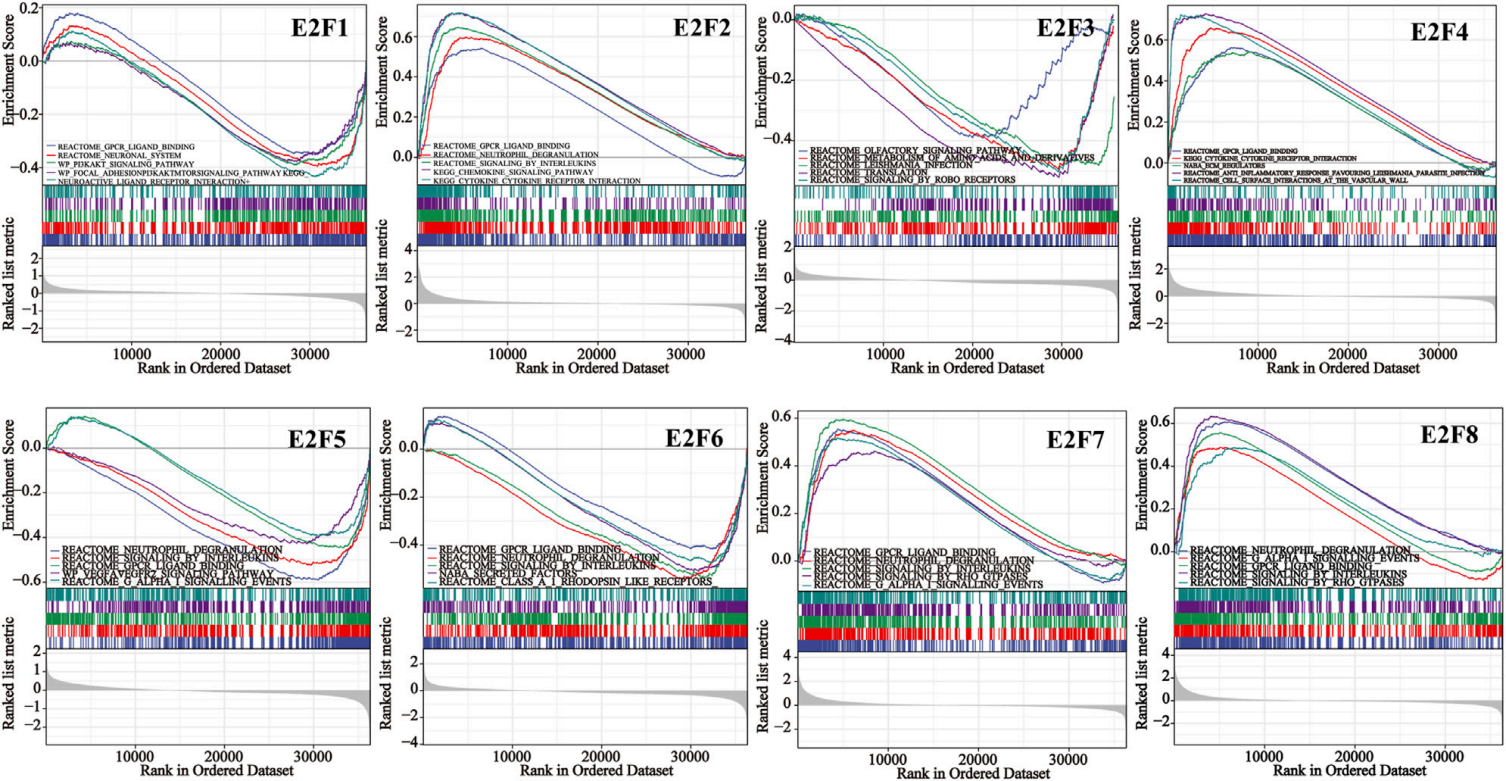
Single-gene GSEA results for each member of the E2F family in THCA.

We conducted ORA utilizing the LinkedOmics database to examine the function of E2Fs in THCA. The outcomes displayed that the F2F family was closely associated with Ras signaling pathway, PDGF signaling pathway, apoptosis, and immune response (Figure 5). The signaling pathways impacted by the expression of the E2F family in THCA were then investigated using single-gene GSEA. The findings showed that the functions of the E2F family may focus on GPCR ligand binding, neutrophil degranulation, and signaling by interleukins (Figure 6).

### 3.5 Relationship between E2F family expression and immunosuppressive agents in THCA

**FIGURE 7:**
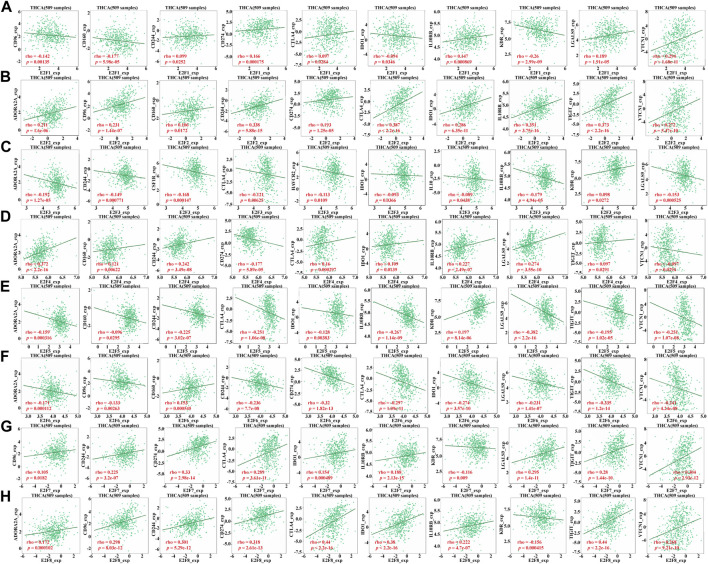
Relationship between E2F family expression and immunosuppressive agents in THCA. **(A–H)** Relationship between E2F1–8 expression and immunosuppressive agents in THCA.

By regulating the activity of related immune cells, the immune checkpoint inhibitor (ICI) improves anti-tumor immune response, which is a major progress in tumor treatment in recent years. Therefore, the TISIDB database was used to explore whether E2Fs in THCA is associated with immune checkpoint inhibitors, for the purpose of exploring whether the function of the E2F family in THCA development is related to the immunosuppressive effect. We included all eight members of the E2F family in this study, and the findings demonstrated that their expression levels correlated differently with each immune checkpoint inhibitor (Figures 7A–H).

### 3.6 Relationship between E2F and immune infiltration in THCA

**FIGURE 8:**
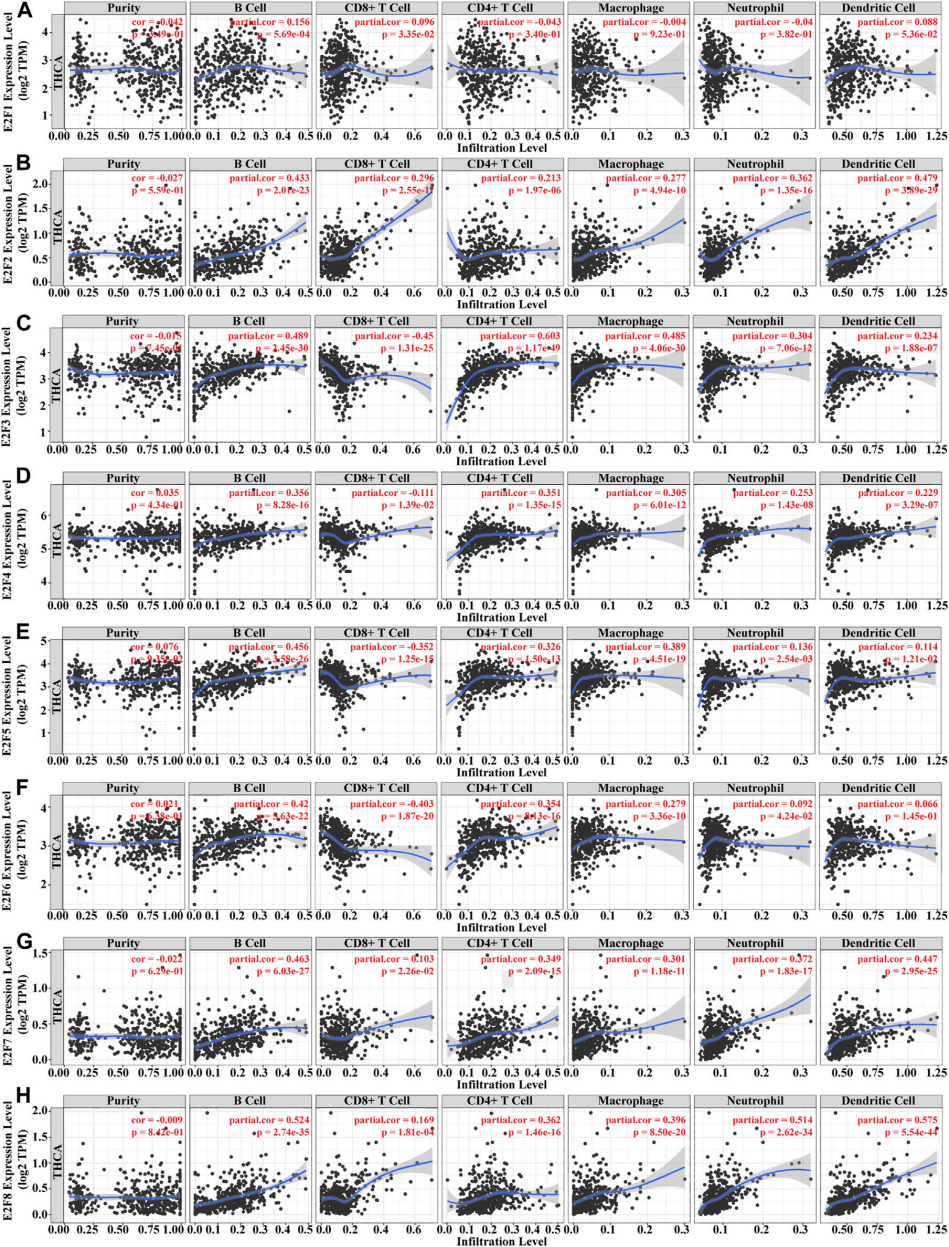
Immune infiltration analysis of the E2F family in THCA. **(A–H)** Association between E2F1–8 levels and immune-infiltrating cells in THCA.

**FIGURE 9:**
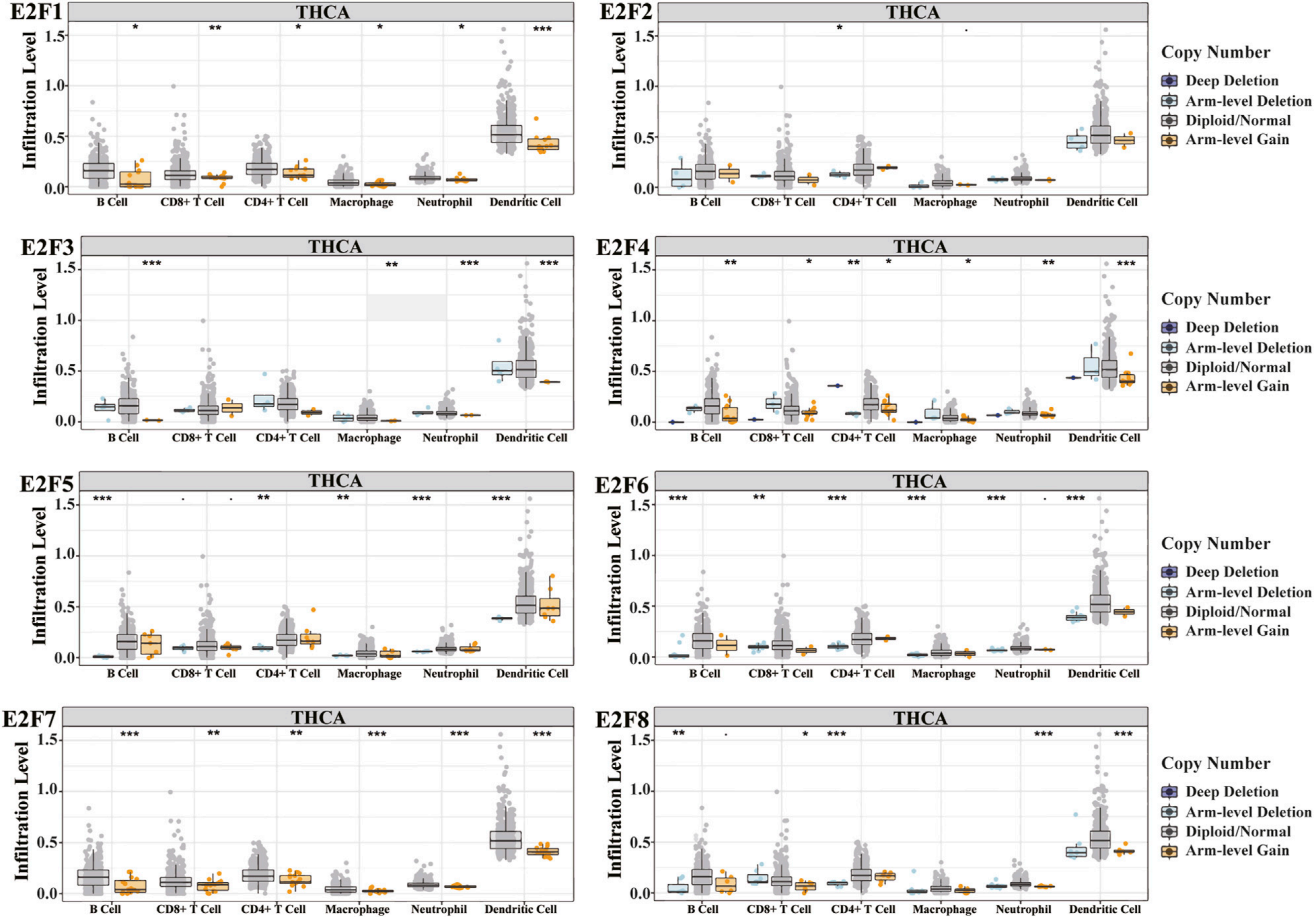
Relationship between the level of cancer infiltration of E2Fs and different somatic copy number alterations in THCA. *p < 0.05; **p < 0.01; ***p < 0.001.

Immune infiltration and cancer microenvironment develop a significant function in the emergence and development of various malignancies. Different clinical outcomes might result from the dynamic interplay of tumor genetic features and tumor microenvironment. We studied the association between the E2F family and tumor-infiltrating lymphocytes (TILs) in THCA. The findings demonstrated that the E2F family members were strongly related to TIL, among which B cells and CD8^+^ T cells were positively correlated with all E2F family members. CD4^+^ T cells, macrophages, and neutrophils were positively correlated with E2F family members except E2F1. Dendritic cells were positively correlated with E2F family members except E2F1 and E2F6 (Figures 8A–H). The SCNA template analysis in the TIMER database showed that the E2F family members’ arm-level deletion and arm-level gain in THCA were strongly related to the level of TIL (Figure 9). Then, we assessed the association between E2F level and numerous immune infiltration-related indicators in order to further investigate the link between tumor-infiltrating immune cells and E2F family members in THCA. The results showed a great association between the level of E2F2, 7, and 8 and most of the ensembles of immune-infiltrating cell markers (Supplementary Table S4). In particular, for the T-cell exhaustion-associated marker ensemble, the outcomes were in line with Figure 7.

## 4 Discussion

THCA is the most prevalent endocrine cancer. The prevalence of THCA is quickly growing worldwide, and most thyroid cancers have a good prognosis. However, some poorly differentiated THCA and high-risk papillary thyroid carcinoma (PTC) are highly aggressive, prone to local invasion and distant metastasis, and there is no specific treatment (Wang et al., 2019; Li et al., 2020). Therefore, elucidating the molecular mechanism of THCA development and progression has important clinical application value for early intervention and prognosis prediction of the disease.

Higher eukaryotes include a family of transcription factors called E2Fs, which may be classified as either transcription activators or transcription repressors depending on their specific roles. Recently, various research studies have evaluated the value of E2F family expression in THCA; however, the study of the key role of E2Fs in THCA is still incomplete (Nie et al., 2022; Yang et al., 2022). In this research, the level of E2Fs in THCA, tumor stage and grade, prognosis, and tumor immunity were analyzed and studied, and comprehensive data were provided for the clinical search for new diagnostic and prognostic markers and therapeutic targets of THCA.

To the best of our knowledge, this research is the first to investigate the level of all E2Fs in pan-cancer and their prognostic values in THCA. Analysis of the pan-cancer data in TCGA project using the R language revealed that eight E2F family members were strongly expressed in various cancers. This is in agreement with the findings of many previous research studies (Garneau et al., 2009; Fang et al., 2018; Moreno et al., 2021). E2F1, 2, 3, 7, and 8 were strongly expressed in THCA. The expression level of the E2F1 protein was substantially higher in THCA than that in normal tissues, according to immunohistochemistry data. Furthermore, expression of E2F family members is associated with race, gender, age, TNM stage, pathological stage, histological type, extrathyroidal extension, and primary neoplasm focus type. A prognostic study revealed that the prognosis was poor in THCA patients who had high levels of E2F2, 7, and 8. However, the subgroup K–M analysis of the association between the expression of E2Fs and the clinicopathology of THCA showed that the high level of E2F1 was associated with poor PFI in patient with THCA in age, T stage, and pathological stage. Finally, in order to find the independent risk variables affecting the prognosis of THCA, we performed a regression analysis. We found that T and M stages, pathologic stage, extrathyroidal extension, E2F2, 7, and 8 were risk factors associated with THCA, but only M stage was an independent risk factor for the prognosis of THCA. In conclusion, the E2F family is a reliable biomarker for THCA prognosis.

To learn more about the role of E2Fs in THCA, ORA and GSEA were performed. ORA findings showed that the expression of E2Fs was closely related to Ras signaling pathway, PDGF signaling pathway, apoptosis, and immune response. The functions of the E2F family may focus on GPCR ligand binding, neutrophil degranulation, and signaling by interleukins by single-gene GSEA. Functional enrichment analysis helps us to study the mechanism of E2Fs in THCA, thus guiding further research directions.

The human immune system is a fine homeostasis management system regulated by a dual signaling system. Costimulatory signaling and coinhibitory signaling are collectively referred to as co-signaling systems or immune checkpoints (Chen and Flies, 2013). CTLA4, the first immune checkpoint, was an important scientific discovery in the 1990s. Researchers have developed monoclonal antibodies against CTLA4, which have shown good efficacy in the treatment of unresectable, metastatic melanoma (Kähler and Hauschild, 2011). Since then, research on the immune checkpoint and its inhibitors has entered a period of rapid development. After 10 years of development, several new immune checkpoints have been discovered, and monoclonal antibody drugs or small-molecule inhibitors against them have been developed successively. Among them, the well-studied immune checkpoints include B- and T-lymphocyte attenuators (BTLAs), TIGIT, and IDO1 (Zhai et al., 2021). In this research, we demonstrated that the E2Fs is significantly related to immune checkpoint inhibitors, but the association is variable. For example, the level of CTLA4 in THCA was positively related to E2F1, 2, 4, 7, and 8 and negatively related to E2F3, 5, and 6. However, for IDO1, its expression in THCA was positively related to E2F2, 4, 7, and 8 and negatively related to E2F1, 3, 5, and 6. This suggests that the underlying mechanism is far from clear and needs further study.

Tumor cells, stromal cells like fibroblasts, immune cells such as T lymphocytes, NK cells, macrophages, and dendritic cells, as well as extracellular matrix containing biochemical substances generated by the aforementioned cells make up the tumor microenvironment (TME) (Shenkman et al., 2018; Roma-Rodrigues et al., 2019; Wei et al., 2021). TME is not only important for tumor proliferation, invasion, and metastasis but also depends on the cross-talk between malignant cells and the surrounding environment, which affects the therapeutic effect (Meurette and Mehlen, 2018; Hinshaw and Shevde, 2019). To further validate the potential association between E2Fs and the immune infiltration in THCA, we explored the association among E2Fs, immune-infiltrating cells, and immune checkpoint gene expression levels. The findings demonstrated that E2F members were strongly related to TIL, among which B cells and CD8^+^ T cells were positively related to all E2F family members. CD4^+^ T cells, macrophages, and neutrophils were positively related to E2F family members except E2F1. Dendritic cells were positively correlated with E2F family members except E2F1 and E2F6. These results suggested that the E2F family may serve as a key immunomodulatory target in THCA progression.

Although this research used a series of databases to conduct a comprehensive analysis of the role of E2Fs in thyroid cancer, it still has some shortcomings. First and foremost, only bioinformatics analytical approaches were employed in this study. Moreover, as the outcomes are drawn solely from public databases, it could result in bias into our findings. Specific experimental and clinical research studies to illustrate the putative function of E2Fs in thyroid cancers are not achievable due to limited experimental circumstances. More research on the mechanism of actions of E2Fs at the cellular and molecular levels will help clarify its function in THCA, which will be the focus of our future research.

The level of eight E2F members in THCA tissues, their association with the clinical stage grade, the prognosis of THCA patients, and immune infiltration were all examined in this work. At the transcriptional level, it was discovered that THCA tissues had significant levels of E2F expression and was highly related to the clinicopathological stage of THCA. The PFI prognosis of THCA patients with a high level of E2F2, E2F7, and E2F8 is poor, suggesting that E2F2, E2F7, and E2F8 may be an independent prognostic biological indicator of THCA. In addition, E2F expression in THCA also increased the level of immune infiltration of CD4^+^ T cells, macrophages, neutrophils, and dendritic cells. Our research offers interesting treatment targets and novel THCA biomarkers.

## Data Availability

The original contributions presented in the study are included in the article/Supplementary Material; further inquiries can be directed to the corresponding authors.
